# Phase-Space Formulation of Shock-Containing Irrotational Barotropic Euler Flow

**DOI:** 10.3390/e28080906

**Published:** 2026-08-13

**Authors:** Sandor M. Molnar, Joseph R. Godfrey

**Affiliations:** 1 Institute of Astronomy and Astrophysics, Academia Sinica, No. 1, Section 4, Roosevelt Road, Taipei 10617, Taiwan; smmolnar@gmail.com; 2 Grado Department of Industrial and Systems Engineering, Virginia Tech, Alexandria, VA 22305, USA

**Keywords:** Koopman–von Neumann, Weyl quantization, Wigner function, Moyal bracket, Euler equation, Burgers equation, shock waves, phase-space methods

## Abstract

We develop a KvN/Weyl/Wigner/Moyal phase-space formulation for shock-containing compressible, irrotational, barotropic Euler flow. Smooth branches are represented by branchwise Wigner distributions, while piecewise-smooth entropy-admissible shocks generate an interface-supported defect in the weak phase-space balance. This defect is concentrated on the moving shock surface and is weighted by the normal relative transport flux between the one-sided branches. An exact planar constant-state three-dimensional example shows how the same mass flux is transferred between distinct velocity-space supports and how its moments recover the classical jump structure. We also introduce a shock solution of the one-dimensional Burgers equation with a triangular initial profile as an exactly solvable reduced benchmark. In this example, the shock trajectory, transported branch weights, branchwise Wigner transforms, and a two-component localized phase-space defect are obtained in closed form. The construction is a restricted branchwise representation of Euler shocks already selected by the Rankine–Hugoniot and entropy conditions; it is not a new admissibility criterion or a complete global Wigner theory across discontinuities. The formulation separates smooth phase-space evolution from singular interface contributions within a unified construction and provides a compact diagnostic description of shock-supported phase-space structure.

## 1. Introduction

Modern theoretical work has revealed structural similarities between classical fluid dynamics and quantum-mechanical phase-space methods. The Euler equations possess a Hamiltonian structure, with both canonical and non-canonical formulations playing an important role in geometric fluid mechanics [1,2,3]. Conversely, the Weyl–Wigner–Moyal formalism represents operator dynamics in terms of Weyl symbols, Wigner functions, and Moyal brackets [4,5,6,7,8,9]. The Koopman–von Neumann (KvN) framework provides a bridge between these viewpoints by lifting classical dynamics to a linear Hilbert-space evolution [10,11,12,13]. Modern Koopman-wavefunction and geometric formulations likewise show that classical dynamics can be expressed in Hilbert-space language without making the dynamics quantum mechanical [14,15].

This paper is related to but distinct from kinetic and measure-theoretic descriptions of shocks. Classical shock theory treats configuration-space discontinuities through Rankine–Hugoniot relations and entropy admissibility criteria [16,17,18,19]. Kinetic and measure-valued formulations encode entropy defects and weak limits by singular or defect measures for scalar conservation laws, isentropic gas dynamics, and related systems [20,21,22,23,24,25,26,27,28,29]. Koopman-based treatments of Burgers dynamics provide a related operator-theoretic viewpoint on the scalar benchmark used below [30].

Our earlier paper [31] introduced a smooth-region KvN/Weyl/Wigner/Moyal formulation for compressible, irrotational, barotropic Euler flow. In the present contribution, we extend the framework introduced in [31] to shock-containing flow. Although the formulation uses mathematical tools commonly associated with quantum mechanics–the KvN, Weyl, Wigner, and Moyal methods–it remains entirely classical. In the present notation, u(r,t) is the physical Eulerian velocity and v is an auxiliary velocity-like phase-space coordinate, a canonical variable conjugate to r, establishing the phase-space coordinates (r,v). The constant α couples spatial displacement with the auxiliary velocity v via a dimensionless Fourier phase within the Wigner kernel. Consequently, α governs the scaling conversion between spatial separation and velocity, thereby setting the resolution of the phase-space representation. Note that α is not associated with Planck’s constant, and the framework requires no small-α asymptotic assumption; again, the formulation operates entirely within classical, not quantum, physics. The Wigner function then provides a phase-space representation of spatial-velocity correlations and coherence at that scale.

The amplitude–phase lift used here is fixed by the continuity and Euler velocity-potential equations. Its Weyl symbol, also known as the Hamiltonian symbol, is

(1) $$H\left(r,v,t\right)=v\cdot u\left(r,t\right)+h\left(\rho \left(r,t\right)\right)-\frac{1}{2}{\left|u\left(r,t\right)\right|}^{2},$$

where *h* is the specific enthalpy. The relation of this expression to, and its correction of, the physical-space symbol used in Ref. [31] is discussed in Appendix A.1. Although H is affine in v, this alone does not truncate the Moyal series when the coefficients are nonlinear in position. General smooth branches obey the full Moyal equation. Exact Moyal-to-Poisson reduction holds when the symbol is at most quadratic in the canonical variables; this includes the constant-state Euler branches and the affine Burgers branches used below.

The question addressed here is how a separately defined branchwise phase-space description is completed when an entropy-admissible Euler solution contains shocks. We assume a piecewise-smooth solution with a sufficiently regular moving shock surface and well-defined one-sided phase-space traces. The shock and its speed are selected first at the fluid level by the Rankine–Hugoniot relations and the entropy condition. We then assemble the one-sided phase-space branches and derive the interface defect generated by the configuration-space flux of the Poisson component. The defect is supported on the moving shock surface and weighted by the normal transport velocity relative to that surface.

The construction is branchwise, treating the flow separately on the two smooth sides of the moving shock. Because the Wigner transform is nonlocal, each one-sided branch is supplied with a specified smooth extension. The resulting piecewise phase-space distribution is not, in general, the exact Wigner transform of a single global wavefunction, which may contain cross-interface interference and other nonlocal contributions. A complete nonlinear analysis of shocks within the KvN/Weyl/Wigner/Moyal framework developed in [31] is beyond the scope of the present work.

In this branchwise setting, we state the smooth-branch Moyal hierarchy for the symbol in Equation (1). We then derive the three-dimensional (3D) interface identity using only well-defined branchwise quantities and one-sided traces. Finally, we present two exact quadratic-symbol examples: a planar constant-state 3D Euler shock and a one-dimensional (1D) Burgers shock generated from a triangular initial profile. The Burgers case is an exactly solvable reduced benchmark in which the two incoming branch traces produce distinct defect supports in velocity space.

For fluid dynamics, the value of this formulation lies not in a new shock condition, but in a phase-space organization of shock transport. It separates smooth evolution within the one-sided flow regions from the singular transfer carried by the moving interface, while its lowest velocity moments recover the Rankine–Hugoniot mass and momentum jump relations. Before those moments are taken, however, the interface defect retains how the shock redistributes phase-space support between the distinct one-sided velocities. This phase-space description captures shock transport details not visible using only configuration-space jump relations.

The paper is organized as follows. Section 2 gives the general smooth-branch Moyal equation and its exact quadratic-symbol reductions. Section 3 develops the branchwise interface identity and the planar constant-state 3D example. Section 4 introduces the triangular-profile Burgers shock as an exactly solvable reduced benchmark and derives its shock trajectory, branchwise Wigner transforms, and localized phase-space defect. Section 5 discusses the physical interpretation, scope, and limitations of the branchwise phase-space formulation. Finally, Section 6 presents our concluding remarks.

## 2. Smooth-Branch Moyal Evolution and Exact Quadratic-Symbol Cases

We consider a compressible, irrotational, barotropic Euler branch with density ρ(r,t), velocity u(r,t)=∇ϕ(r,t), and pressure P(ρ). Throughout this construction, ρ and u are assumed given solutions of the irrotational barotropic Euler equations on each smooth branch. The auxiliary amplitude–phase wavefunction and its Wigner representation are constructed from these Euler fields; the wave equation is not introduced as an independent closed evolution for determining them. The specific enthalpy h(ρ) is defined by

(2) $$\nabla h\left(\rho \right)=\frac{1}{\rho }\nabla P\left(\rho \right).$$

After fixing the irrelevant time-dependent gauge of ϕ, the continuity and Euler velocity-potential equations are

(3) $$\partial_{t}\rho +\nabla_{r}\cdot \left(\rho u\right)=0,$$

and

(4) $$\partial_{t}\phi +\frac{1}{2}{\left|u\right|}^{2}+h\left(\rho \right)=0.$$

Define the amplitude–phase lift

(5) $$\psi \left(r,t\right)=\sqrt{\rho (r,t)}\exp\;\left(\frac{i\phi (r,t)}{\alpha }\right),$$

where $\left[\alpha \right]=L^{2}/T$. Direct substitution of Equations (3) and (4) gives

(6) $$i\alpha \;\partial_{t}\psi ={\hat{H}}_{u}\psi ,$$

with the Weyl-ordered branch evolution operator

(7) $${\hat{H}}_{u}=\frac{1}{2}\left[u\left(\hat{r},t\right)\cdot \hat{v}+\hat{v}\cdot u\left(\hat{r},t\right)\right]+h\left(\rho \right)-\frac{1}{2}{\left|u\right|}^{2},\;\hat{v}=-i\alpha \nabla_{r}.$$

In coordinate representation,

(8) $${\hat{H}}_{u}=-i\alpha \left(u\cdot \nabla_{r}+\frac{1}{2}\nabla_{r}\cdot u\right)+h\left(\rho \right)-\frac{1}{2}{\left|u\right|}^{2}.$$

Its Hamiltonian symbol is

(9) $$H\left(r,v,t\right)=v\cdot u\left(r,t\right)+G\left(r,t\right),\;G=h\left(\rho \right)-\frac{1}{2}{\left|u\right|}^{2}.$$

The scalar term G is required by the Euler velocity-potential equation. Retaining only v·u generates passive transport of a square-root density but does not reproduce the phase evolution of Equation (5).

### Wigner Convention and Smooth-Branch Moyal Evolution

We use the α-scaled Wigner convention

(10) $$W\left(r,v,t\right)=\int \frac{d^{3}y}{{\left(2\pi \alpha \right)}^{3}}\exp\;\left(-\frac{i}{\alpha }v\cdot y\right)\psi^{*}\;\left(r-\frac{y}{2},t\right)\psi \;\left(r+\frac{y}{2},t\right).$$

Here v has the dimensions of velocity. For a plane wave or the specific spatially uniform-amplitude quadratic-phase extensions used in the exact examples below, this normalization places the velocity-space support directly at v=∇ϕ. For a general finite-α branch, the Wigner transform is nonlocal and need not be monokinetic.

For branch symbols and Wigner objects for which the Weyl star products are defined, the exact smooth-branch Wigner evolution is

(11) $$\partial_{t}W={\left\{H,W\right\}}_{M}=\frac{2}{\alpha }\;H\sin\;\left(\frac{\alpha }{2}\Lambda \right)W,$$

where

(12) $$\Lambda ={\overset{\leftarrow }{\nabla }}_{r}\cdot {\vec{\nabla }}_{v}-{\overset{\leftarrow }{\nabla }}_{v}\cdot {\vec{\nabla }}_{r}.$$

Equation (11) is understood in the standard Weyl-calculus sense on each smooth branch. The derivative expansion below is formal, or asymptotic, unless stronger symbol-class or analyticity assumptions justify the series term by term.

Using index notation with summation over repeated indices, the first terms are

(13) $$\begin{array}{cc} \partial_{t}W= & \left[v_{j}\left(\partial_{i}u_{j}\right)+\partial_{i}G\right]\partial_{v_{i}}W-u_{i}\partial_{r_{i}}W\\ \; & -\frac{\alpha^{2}}{24}[\left(v_{\ell }\partial_{i}\partial_{j}\partial_{k}u_{\ell }+\partial_{i}\partial_{j}\partial_{k}G\right)\partial_{v_{i}}\partial_{v_{j}}\partial_{v_{k}}W\\ \; & \;-3\left(\partial_{i}\partial_{j}u_{k}\right)\partial_{v_{i}}\partial_{v_{j}}\partial_{r_{k}}W]+O\left(\alpha^{4}\right). \end{array}$$

Thus, affinity in v does not remove the mixed derivatives involving higher spatial derivatives of u, nor does it remove the spatial derivatives of G.

Define the Poisson force

(14) $$F\left(r,v,t\right)=-\nabla_{r}H=-{\left(\nabla u\right)}^{\;⊤}v-\nabla G=-{\left(\nabla u\right)}^{\;⊤}\left(v-u\right)-\nabla h.$$

The final equality uses ${\nabla (|u|}^{2}{/2)=\left(\nabla u\right)}^{⊤}u$. The Poisson transport operator is

(15) $$L_{P}W=\partial_{t}W+\nabla_{r}\cdot \left(uW\right)+\nabla_{v}\cdot \left(FW\right).$$

The exact Moyal equation may then be written as

(16) $$L_{P}W=C_{M}\left[W;u,\rho \right],$$

where $C_{M}$ is the higher-order Moyal derivative contribution, formed by the terms proportional to $\alpha^{2}$ and higher in the formal Moyal derivative expansion of Equation (13). No small-α approximation is assumed.

Exact Moyal-to-Poisson reduction for arbitrary *W* occurs when the symbol H is at most quadratic in the canonical variables (r,v). A sufficient condition is

(17) u(r,t)=A(t)r+b(t),G(r,t)isatmostquadraticinr.

Then $C_{M}=0$ and

(18) $$L_{P}W=0$$

holds exactly. The planar constant-state Euler branches satisfy this condition. The affine Burgers branches also satisfy it for their scalar symbol $H_{B}=vu-u^{2}/2$, derived in Section 4.3.

## 3. Shock-Supported Interface Defect in the Branchwise Phase-Space Formulation

### 3.1. Branchwise Setting and Mathematical Scope

Let the shock surface be represented locally by

(19) $$\Sigma_{t}=\left\{r:\chi \left(t,r\right)=0\right\},\;n=\frac{\nabla_{r}\chi }{|\nabla_{r}\chi |},\;V_{n}=-\frac{\partial_{t}\chi }{|\nabla_{r}\chi |}.$$

We assume that χ is $C^{1}$ with $|\nabla_{r}\chi |\neq 0$ near the interface, that the Euler branches $\left(\rho_{\pm },u_{\pm }\right)$ are smooth up to their respective sides, and that the associated phase-space branches $W_{\pm }$ possess one-sided traces on $\Sigma_{t}$ as tempered distributions, or finite Radon measures, in v. Where the full higher-order Moyal derivative contribution is retained, we additionally assume that the separate branch symbols and Wigner objects lie in a Weyl-calculus class for which the branchwise star products are defined.

Because the Wigner transform is nonlocal, a one-sided wavefunction does not uniquely determine a finite-α Wigner object without an extension across the boundary of its domain. Choose specified smooth extensions ${\tilde{\psi }}_{\pm }$ of the one-sided amplitude–phase branches and define

(20) $$W_{\pm }\left(r,v,t\right)=\int \frac{d^{3}y}{{\left(2\pi \alpha \right)}^{3}}\exp\;\left(-\frac{i}{\alpha }v\cdot y\right){\tilde{\psi }}_{\pm }^{*}\;\left(r-\frac{y}{2},t\right){\tilde{\psi }}_{\pm }\;\left(r+\frac{y}{2},t\right).$$

More generally, $W_{\pm }$ may be separately specified phase-space branches satisfying the equations below. In either case, we assume the one-sided traces

(21) $$W_{\pm }\left(r_{\Sigma },v,t\right)=\lim_{r\to r_{\Sigma },\;\pm \chi \left(t,r\right)>0}W_{\pm }\left(r,v,t\right),\;r_{\Sigma }\in \Sigma_{t},$$

exist in the stated distributional sense. In the exact examples, the natural constant-state or affine extension fixes the construction explicitly.

The branchwise assembled object is

(22) $$W_{\mathrm{br}}\left(r,v,t\right)=W_{-}\left(r,v,t\right)\Theta \left(-\chi \right)+W_{+}\left(r,v,t\right)\Theta \left(\chi \right).$$

Equation (22) is not asserted to equal the exact global Wigner transform of a single wavefunction formed by joining the two one-sided branches across the interface. Such a transform can contain nonlocal cross-interface interference terms and can depend on the global extension of the two branches. The construction below isolates the weak interface defect of the specified branchwise objects.

The result is therefore a restricted trace-admitting construction. The identity derived below is a formal weak identity under the stated branch regularity and trace assumptions rather than a general functional-analytic theorem for arbitrary weak Euler solutions. It is not a universal Wigner closure relation or a shock-admissibility principle; the entropy-admissible shock and its traces are selected first at the fluid level.

### 3.2. Branchwise Moyal Hierarchy and Poisson Interface Identity

Define

(23) $$u_{n,\pm }=u_{\pm }\cdot n,\;Q_{\pm }=\left\{\left(t,r\right):\pm \chi \left(t,r\right)>0\right\}.$$

On each smooth branch, let

(24) $$L_{P,\pm }W_{\pm }=\partial_{t}W_{\pm }+\nabla_{r}\cdot \left(u_{\pm }W_{\pm }\right)+\nabla_{v}\cdot \left(F_{\pm }W_{\pm }\right)=C_{M,\pm },$$

where

(25) $$F_{\pm }=-{\left(\nabla u_{\pm }\right)}^{\;⊤}v-\nabla G_{\pm },\;G_{\pm }=h\left(\rho_{\pm }\right)-\frac{1}{2}{\left|u_{\pm }\right|}^{2}.$$

All derivatives in Equations (24) and (25) are taken only inside the smooth branches. No product between a surface-distributional derivative of the Euler fields and $W_{\mathrm{br}}$ is formed.

Define the branchwise higher-order Moyal derivative contribution

(26) $$C_{M}^{\mathrm{br}}=C_{M,-}\Theta \left(-\chi \right)+C_{M,+}\Theta \left(\chi \right).$$

The distributional differentiation of Equation (22) gives

(27) $$L_{P}^{\mathrm{br}}W_{\mathrm{br}}+\mu_{\Sigma }^{P}=C_{M}^{\mathrm{br}},$$

Equation (27) is assembled from the separately evaluated smooth-branch Moyal equations and the distributional Poisson flux of the Heaviside factors. It is not obtained by applying a full global Moyal operator to $W_{\mathrm{br}}$.

The Poisson interface defect is defined by

(28) $$\begin{array}{cc} \langle \mu_{\Sigma }^{P},\varphi \rangle =\int_{R}\int_{\Sigma_{t}}\int_{R^{3}}[\; & \left(u_{n,-}-V_{n}\right)W_{-}\varphi \\ \; & -\left(u_{n,+}-V_{n}\right)W_{+}\varphi ]\;d^{3}v\;dS\;dt \end{array}$$

for every $\varphi \in C_{c}^{\infty }\left(R_{t}\times R_{r}^{3}\times R_{v}^{3}\right)$. The sign follows from the convention in Equation (22). The coefficient of each trace is the normal configuration-space transport velocity relative to the moving interface. Tangential motion does not generate a crossing contribution.

When the branch symbols are quadratic, or when the Moyal hierarchy is truncated to its Poisson component, $C_{M,\pm }=0$. Equation (27) then reduces to

(29) $$L_{P}^{\mathrm{br}}W_{\mathrm{br}}+\mu_{\Sigma }^{P}=0.$$

Equivalently, branchwise integration by parts gives

(30) $$\begin{array}{cc} \; & \sum_{\sigma =\pm }\int_{Q_{\sigma }}\int_{R^{3}}W_{\sigma }\left[\partial_{t}\varphi +u_{\sigma }\cdot \nabla_{r}\varphi +F_{\sigma }\cdot \nabla_{v}\varphi \right]\;d^{3}v\;d^{3}r\;dt\\ \; & \;=\langle \mu_{\Sigma }^{P},\varphi \rangle . \end{array}$$

For nonlinear branches, Equation (27) retains the separately computed smooth-branch higher-order Moyal derivative contributions. A genuinely global Moyal construction can additionally contain higher interface distributions and nonlocal cross-interface terms.

### 3.3. Monokinetic Phase-Space Example and Rankine–Hugoniot Interpretation

A useful branchwise monokinetic Wigner representation is

(31) $$W_{\pm }\left(r_{\Sigma },v,t\right)=\rho_{\pm }\left(r_{\Sigma },t\right)\delta^{\left(3\right)}\;\left(v-u_{\pm }\left(r_{\Sigma },t\right)\right).$$

At finite α, Equation (31) is not asserted to be the exact Wigner trace of an arbitrary nonlinear one-sided branch. It is exact for the global plane-wave extension and for the specific spatially uniform-amplitude quadratic-phase extensions used in the explicit examples below; otherwise, it may be read as a monokinetic phase-space ansatz.

The shock data are inherited from an entropy-admissible weak solution of the barotropic Euler equations. With $\left[f\right]=f_{+}-f_{-}$, the normal Rankine–Hugoniot conditions are

(32) $$V_{n}\left[\rho \right]=\left[\rho u_{n}\right],$$

and

(33) $$V_{n}\left[\rho u_{n}\right]=\left[\rho u_{n}^{2}+P\left(\rho \right)\right].$$

These relations, together with the entropy condition, determine the admissible shock states and speed; the phase-space defect does not select them.

Using the branchwise Wigner functions defined in Equation (31), Equation (28) becomes

(34) $$\begin{array}{cc} \langle \mu_{\Sigma }^{P},\varphi \rangle =\int_{R}\int_{\Sigma_{t}} & \left[(u_{n,-}-V_{n}\right)\rho_{-}\varphi \left(t,r,u_{-}\right)\\ \; & -\left(u_{n,+}-V_{n}\right)\rho_{+}\varphi \left(t,r,u_{+}\right)]\;dS\;dt. \end{array}$$

Thus, the defect records the normal relative mass transport between two velocity-space supports. The density–pressure coupling remains encoded in the fluid-level Euler jump data.

### 3.4. Exact Planar Constant-State 3D Shock

Let

(35) $$\chi \left(t,r\right)=n\cdot r-V_{n}t,\;\left(\rho_{-},u_{-}\right),\;\left(\rho_{+},u_{+}\right)=\mathrm{constants},$$

and assume that the two states satisfy the barotropic Euler Rankine–Hugoniot and entropy conditions. The common shock-frame mass flux is

(36) $$j=\rho_{-}\left(u_{n,-}-V_{n}\right)=\rho_{+}\left(u_{n,+}-V_{n}\right).$$

A global plane-wave extension of each constant branch gives the exact branchwise Wigner functions; the corresponding Wigner-transform calculation is given in Appendix A.2.

(37) $$W_{\pm }\left(r,v,t\right)=\rho_{\pm }\delta^{\left(3\right)}\;\left(v-u_{\pm }\right).$$

Because $u_{\pm }$, $\rho_{\pm }$, and $G_{\pm }$ are constant, the symbols are affine in v and all higher Moyal terms vanish exactly. For compact notation, define the surface delta distribution by $\delta_{\Sigma }=\delta \left(\chi \right)\left|\nabla_{r}\chi \right|$; for the planar level set in Equation (35), $|\nabla_{r}\chi |=1$ and hence $\delta_{\Sigma }=\delta \left(\chi \right)$. Substitution into Equation (28) gives

(38) $$\mu_{\Sigma }^{P}=j\;\delta_{\Sigma }\left[\delta^{\left(3\right)}\;\left(v-u_{-}\right)-\delta^{\left(3\right)}\;\left(v-u_{+}\right)\right].$$

Its zeroth velocity-space moment vanishes,

(39) $$\int_{R^{3}}\mu_{\Sigma }^{P}\;d^{3}v=0,$$

whereas its first moment is

(40) $$\int_{R^{3}}v\;\mu_{\Sigma }^{P}\;d^{3}v=j\left(u_{-}-u_{+}\right)\delta_{\Sigma }=\left[P\right]\;n\;\delta_{\Sigma },$$

where $\left[P\right]=P_{+}-P_{-}$ and the final equality follows from the vector Euler momentum jump condition. Thus, the same mass flux enters and leaves the shock, but it is transferred between distinct velocity-space supports. The pressure jump is inherited from the prescribed Euler shock states; it is not generated independently by the phase-space construction.

**Figure 1:**
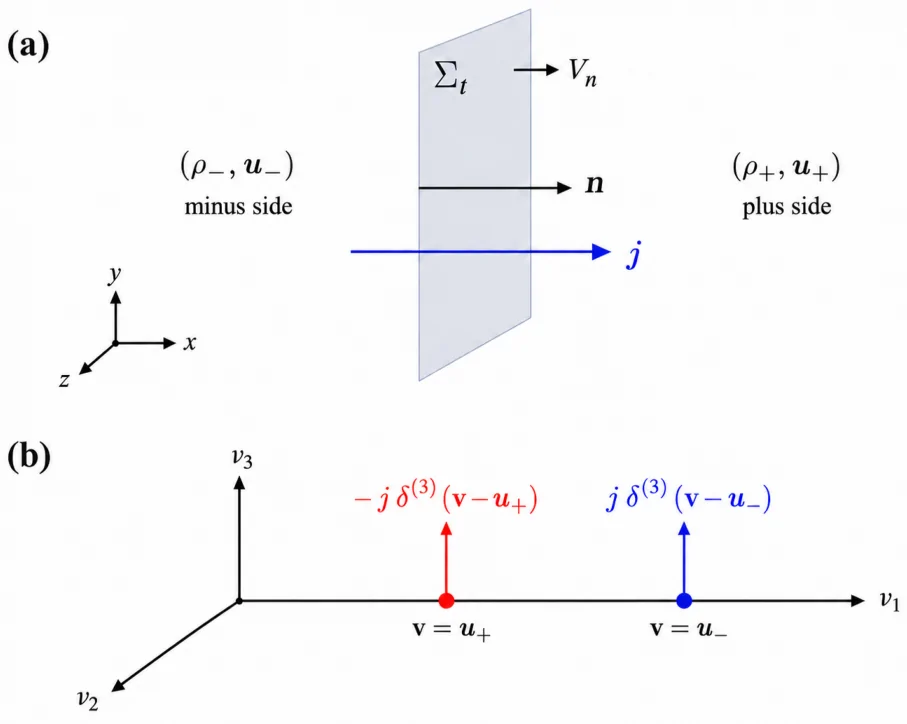
Exact planar constant-state shock example. (**a**) A planar shock surface $\Sigma_{t}$ separates the constant states $\left(\rho_{-},u_{-}\right)$ and $\left(\rho_{+},u_{+}\right)$. The unit normal n points from the minus side to the plus side, $V_{n}$ is the normal shock speed, and *j* is the common shock-frame mass flux. (**b**) The corresponding branchwise velocity-space representation is shown in a normal-flow frame. The axes $v_{1}$, $v_{2}$, and $v_{3}$ are the Cartesian coordinates of the auxiliary velocity space, with $v_{1}$ aligned with the shock normal. The two monokinetic supports are located at $v=u_{-}$ and $v=u_{+}$, with opposite signed contributions to the Poisson-order interface defect. The explicit defect formula and its zeroth- and first-moment relations are given by Equations (38)–(40).

Figure 1 summarizes this exact constant-state example in both physical space and velocity space. Panel (a) shows the planar shock geometry, including the interface $\Sigma_{t}$, the unit normal n, the normal shock speed $V_{n}$, and the common shock-frame mass flux *j*. Panel (b) shows the two branchwise monokinetic supports at $v=u_{-}$ and $v=u_{+}$ together with their opposite signed contributions to the Poisson-order interface defect. The explicit defect formula and its zeroth- and first-moment relations are given by Equations (38)–(40).

The planar example is exact within the explicitly specified branchwise plane-wave extension and provides a 3D counterpart to the affine Burgers benchmark developed next.

## 4. Explicit Benchmark: 1D Burgers Triangular Shock

We demonstrate this mechanism using the 1D inviscid Burgers equation,

(41) $$u_{t}+u\;u_{x}=0.$$

The benchmark is intended to illustrate the interface-source mechanism in an exactly solvable setting, not to justify the 3D interface statement, which was formulated independently in Section 3.

We consider the triangular initial condition

(42) u(x,0)=1−|x|(|x|<1),u(x,0)=0otherwise,

with x∈R and t≥0. For 0<t<1, the classical solution is the piecewise triangular profile

(43) $$u\left(x,t\right)=\left\{\begin{array}{cc} \frac{1+x}{1+t}, & -1<x<t,\\ \frac{1-x}{1-t}, & t<x<1,\\ 0, & \mathrm{otherwise}. \end{array}\right.$$

This pre-shock solution already displays the essential nonlinear steepening of the right branch. As $t\to 1^{-}$, the right lobe sharpens and all of its characteristics converge at the single point (t,x)=(1,1), where the classical solution becomes multivalued and an entropy-admissible shock must be selected.

**Figure 2:**
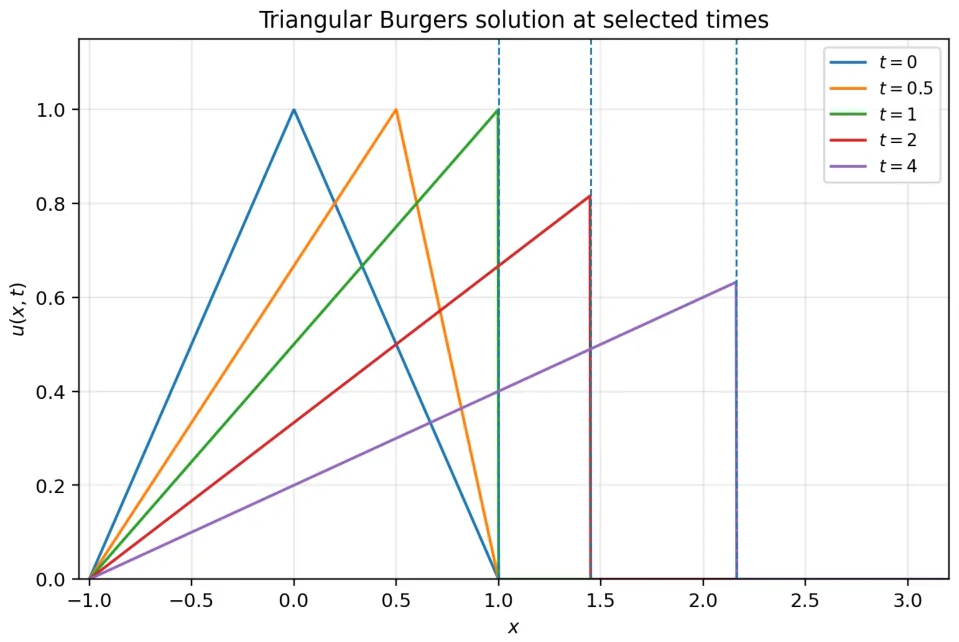
Profiles of the triangular solution at successive times. Nonlinear steepening of the two linear branches produces a cusp at (t,x)=(1,1), where all right-branch characteristics converge and the entropy solution forms a shock. The vertical dashed lines at x=s(t) mark the shock location. For t>1, the left-state velocity decreases as the shock propagates to the right, separating the interior flow from the zero-velocity state ahead of it.

The triangular benchmark retains the features needed for the present phase-space illustration: characteristic crossing, shock formation, entropy selection, and explicit post-shock decay. Figure 2 illustrates the evolution of the triangular profile through shock formation and its subsequent propagation.

### 4.1. Shock Formation and Entropy-Admissible Propagation

The initial profile in Equation (42) consists of two linear branches meeting at x=0. Each point propagates along a characteristic $x\left(t\right)=x_{0}+t\;u\left(x_{0},0\right)$. On the right lobe, where $0<x_{0}<1$ and $u\left(x_{0},0\right)=1-x_{0}$, one has

(44) $$x\left(t\right)=x_{0}+t\;\left(1-x_{0}\right),\;u\left(x\left(t\right),t\right)=1-x_{0},\;\left(0<x_{0}<1\right).$$

All such characteristics arrive simultaneously at x=1 when t=1. Hence the classical solution develops a cusp at (t,x)=(1,1), and the entropy solution forms a shock there.

Let

(45) Γ={(t,x):x=s(t)}

be the shock curve for t≥1, and let $u_{L}\left(t\right)$ and $u_{R}\left(t\right)$ be the left and right traces at x=s(t). For Burgers equation, the Rankine–Hugoniot speed is

(46) $$s^{\prime }\left(t\right)=\frac{f\left(u_{L}\right)-f\left(u_{R}\right)}{u_{L}-u_{R}}=\frac{u_{L}+u_{R}}{2},\;f\left(u\right)=\frac{1}{2}u^{2}.$$

**Figure 3:**
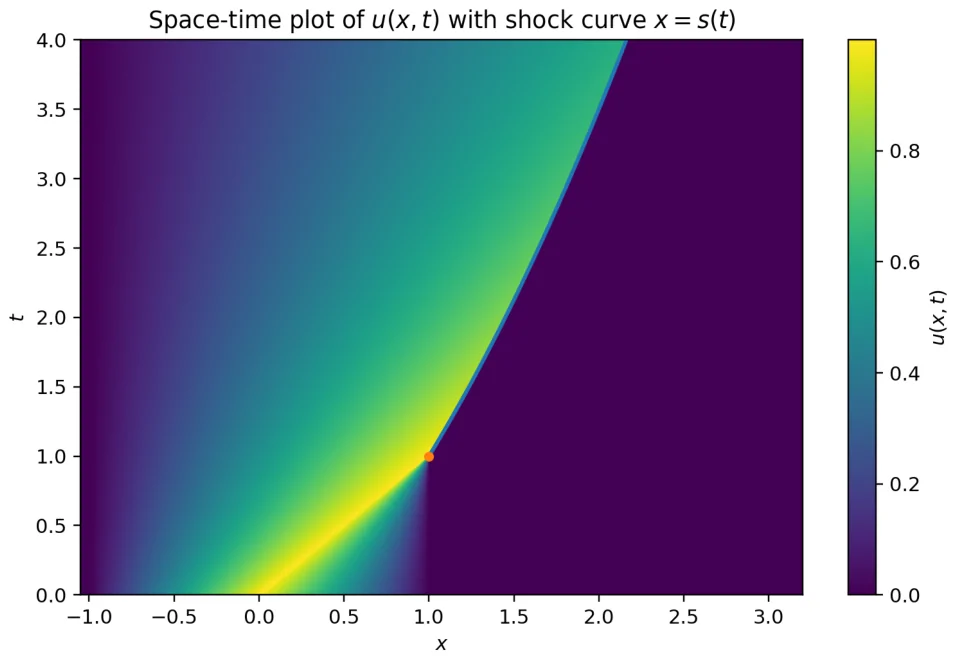
Space–time evolution of the triangular solution. The color map shows u(x,t). The blue curve x=s(t) marks the shock trajectory for t≥1, while the orange dot marks the shock-formation point (t,x)=(1,1), where the characteristics first collide. The shock separates the decaying left state from the zero-velocity state on the right, and its rightward motion reflects the Rankine–Hugoniot propagation governing the entropy-admissible solution.

Here $u_{R}=0$, so Equation (46) gives $s^{\prime }=u_{L}/2$. The Lax condition $u_{L}>s^{\prime }>u_{R}=0$ is therefore satisfied, with characteristics incoming from both sides in the shock frame. Thus, the shock generated at (t,x)=(1,1) is the entropy-admissible weak continuation of the pre-shock triangular profile. Figure 3 shows the corresponding space–time evolution and the shock trajectory x=s(t).

### 4.2. Explicit Post-Shock Solution

For t>1, the shock is reached by characteristics originating from the left lobe, −1<y<0, where the initial velocity is

(47) $$u_{0}\left(y\right)=1+y,\;x\left(t\right)=y+t\;\left(1+y\right).$$

Let y(t)∈(−1,0) label the characteristic that reaches the shock at time *t*. The capture condition, left trace, and Rankine–Hugoniot speed are

(48) $$\begin{array}{cccccc} s(t) & =y(t)+t[1+y(t)],\; & u_{L}\left(t\right) & =1+y(t),\; & s^{\prime }\left(t\right) & =\frac{u_{L}\left(t\right)}{2}, \end{array}$$

with $u_{R}\equiv 0$ and s(1)=1. Differentiating the capture condition and eliminating $s^{\prime }$ gives

(49) $$y^{\prime }\left(t\right)=-\frac{1+y(t)}{2\;(1+t)}.$$

Imposing the entropy-matching condition $u_{L}\left(1^{+}\right)=1$—or equivalently, $y(1^{+})=0$—yields

(50) $$u_{L}\left(t\right)=1+y\left(t\right)=\frac{\sqrt{2}}{\sqrt{\;1+t\;}},$$

and therefore

(51) $$s^{\prime }\left(t\right)=\frac{u_{L}\left(t\right)}{2}=\frac{1}{\sqrt{2}}\frac{1}{\sqrt{\;1+t\;}}.$$

Integrating Equation (51) with s(1)=1 gives the explicit shock trajectory

(52) $$s\left(t\right)=\sqrt{2}\;\sqrt{\;1+t\;}-1,\;t\geq 1.$$

Hence, for t≥1, the entropy solution is

(53) $$u\left(x,t\right)=\frac{1+x}{\;1+t\;}\;\mathrm{for}\;-1<x<s\left(t\right),\;u\left(x,t\right)=0\;\mathrm{for}\;x\geq s\left(t\right).$$

Substitution of Equation (52) into the interior branch verifies $u\left(s{\left(t\right)}^{-},t\right)=\sqrt{2}/\sqrt{1+t}=u_{L}\left(t\right)$. Away from the shock curve Γ, the post-shock solution remains smooth. At the shock, the jump produces the localized phase-space contribution derived below, the 1D counterpart of $\mu_{\Sigma }^{P}$ in Section 3.

### 4.3. Phase-Space Description and the Shock-Supported Source

To construct an amplitude–phase lift of the Burgers solution, we introduce an auxiliary nonnegative density ϱ(x,t) satisfying the equation,

(54) $$\partial_{t}\varrho +\partial_{x}\left(\varrho u\right)=0.$$

This auxiliary density is used to construct the KvN/Wigner amplitude; it is not an additional dynamical variable of the scalar Burgers equation. It may also be interpreted as a pressureless-fluid density if the Burgers equation is embedded in the corresponding continuity–momentum system. Here it serves only as a branchwise Wigner weight. Let $u=\partial_{x}\phi$, and fix the potential gauge by

(55) $$\partial_{t}\phi +\frac{1}{2}u^{2}=0.$$

Then $\psi =\sqrt{\varrho }\exp\left(i\phi /\alpha \right)$ satisfies

(56) $$i\alpha \;\partial_{t}\psi ={\hat{H}}_{B}\psi ,$$

with

(57) $${\hat{H}}_{B}=\frac{1}{2}\left[u\left(\hat{x},t\right)\hat{v}+\hat{v}\;u\left(\hat{x},t\right)\right]-\frac{1}{2}u^{2},\;\hat{v}=-i\alpha \partial_{x}.$$

The corresponding Hamiltonian symbol is therefore

(58) $$H_{B}\left(x,v,t\right)=v\;u\left(x,t\right)-\frac{1}{2}u{\left(x,t\right)}^{2}.$$

The scalar term $-u^{2}/2$ is essential: it makes the monokinetic sheet v=u invariant under the Poisson flow. The associated canonical equations are

(59) $$\dot{x}=u,\;\dot{v}=-\left(v-u\right)u_{x}.$$

On every smooth branch of the triangular solution, u(x,t) is affine in *x*, so Equation (58) is at most quadratic in (x,v). The full Moyal bracket therefore reduces exactly to the Poisson bracket, and the branchwise Wigner function satisfies

(60) $$\partial_{t}W_{\psi }+\partial_{x}\left(uW_{\psi }\right)+\partial_{v}\;\left[-\left(v-u\right)u_{x}W_{\psi }\right]=0$$

away from the shock.

For completeness, the two pre-shock affine extensions may be lifted with spatially uniform transported densities. We choose the same arbitrary initial normalization $\varrho_{0}>0$ for both extensions and let

(61) $$\begin{array}{cccccc} u_{-}^{\mathrm{ext}}\left(x,t\right) & =\frac{1+x}{1+t},\; & \varrho_{-}^{\mathrm{ext}}\left(t\right) & =\frac{\varrho_{0}}{1+t},\; & \phi_{-}^{\mathrm{ext}}\left(x,t\right) & =\frac{{\left(1+x\right)}^{2}}{2(1+t)},\\ u_{+}^{\mathrm{ext}}\left(x,t\right) & =\frac{1-x}{1-t},\; & \varrho_{+}^{\mathrm{ext}}\left(t\right) & =\frac{\varrho_{0}}{1-t},\; & \phi_{+}^{\mathrm{ext}}\left(x,t\right) & =-\frac{{\left(1-x\right)}^{2}}{2(1-t)}. \end{array}$$

Each pair satisfies the auxiliary continuity Equation (54) and the Hamilton–Jacobi Equation (55). The corresponding globally extended wavefunctions and exact branchwise Wigner transforms are

(62) $$\psi_{\pm }^{\mathrm{ext}}=\sqrt{\varrho_{\pm }^{\mathrm{ext}}}\;\exp\;\left(\frac{i}{\alpha }\phi_{\pm }^{\mathrm{ext}}\right),\;W_{\psi ,\pm }^{\mathrm{ext}}\left(x,v,t\right)=\varrho_{\pm }^{\mathrm{ext}}\left(t\right)\delta \;\left(v-u_{\pm }^{\mathrm{ext}}\left(x,t\right)\right).$$

These formulas describe separate global affine extensions, not a single wavefunction formed by joining the two affine branches. The growth $\varrho_{+}^{\mathrm{ext}}\propto {\left(1-t\right)}^{-1}$ as $t\to 1^{-}$ records the vanishing characteristic Jacobian at crossing.

Recall that the shock curve is expressed as Γ={(t,x):x=s(t)} (Equation (45)). We define the incoming branch by the global affine extension

(63) $$u_{L}^{\mathrm{ext}}\left(x,t\right)=\frac{1+x}{1+t},\;\varrho_{L}^{\mathrm{ext}}\left(x,t\right)=\frac{\varrho_{0}}{1+t},$$

where the same constant $\varrho_{0}$ fixes the arbitrary normalization of the auxiliary transported density. We use the exact Wigner transform of this extension before restricting the resulting phase-space branch to x<s(t). On the right, we use the stationary branch

(64) $$u_{R}\left(x,t\right)=0,\;\varrho_{R}\left(x,t\right)=\varrho_{0},\;\phi_{R}\left(x,t\right)=0,\;W_{\psi ,R}\left(x,v,t\right)=\varrho_{0}\;\delta \left(v\right).$$

Thus the branchwise construction uses nonzero smooth transported-density branches on both sides of the shock. This specifies the extension convention and avoids identifying the branchwise object with the global Wigner transform of a spatially truncated wavefunction.

The assembled object is

(65) $$W_{\psi }\left(x,v,t\right)=W_{\psi ,L}\left(x,v,t\right)\Theta \left(s\left(t\right)-x\right)+W_{\psi ,R}\left(x,v,t\right)\Theta \left(x-s\left(t\right)\right).$$

Distributional differentiation gives

(66) $$\partial_{t}W_{\psi }+\partial_{x}\left(uW_{\psi }\right)+\partial_{v}\;\left[-\left(v-u\right)u_{x}W_{\psi }\right]+\mu_{\Gamma }=0,$$

where

(67) $$\begin{array}{cc} \langle \mu_{\Gamma },\varphi \rangle =\int_{1}^{\infty }\;\int_{R} & \left[(u_{L}-s^{\prime }\right)W_{\psi ,L}\left(s\left(t\right),v,t\right)\\ \; & -\left(u_{R}-s^{\prime }\right)W_{\psi ,R}\left(s\left(t\right),v,t\right)]\varphi \left(t,s\left(t\right),v\right)\;dv\;dt \end{array}$$

for every $\varphi \in C_{c}^{\infty }\left(R_{t}\times R_{x}\times R_{v}\right)$. The velocity-space force does not alter the interface coefficient because it differentiates only with respect to *v*.

The exact Wigner transform of the affine extension in Equation (63) is

(68) $$W_{\psi ,L}\left(x,v,t\right)=\frac{\varrho_{0}}{1+t}\delta \;\left(v-\frac{1+x}{1+t}\right),$$

as derived in Appendix A.3. Hence

(69) $$W_{\psi ,L}\left(s{\left(t\right)}^{-},v,t\right)=\varrho_{L}\left(t\right)\delta \left(v-u_{L}\left(t\right)\right),$$

with

(70) $$\varrho_{L}\left(t\right)=\frac{\varrho_{0}}{1+t},\;u_{L}\left(t\right)=\frac{\sqrt{2}}{\sqrt{1+t}},\;s^{\prime }\left(t\right)=\frac{u_{L}\left(t\right)}{2}.$$

This delta support is exact for the specified global affine extension. It is not asserted to be the exact Wigner transform of the physically truncated post-shock branch or a general closure for non-affine or multibranch Burgers solutions [25,27,32,33]. The stationary right branch contributes the exact trace

(71) $$W_{\psi ,R}\left(s{\left(t\right)}^{+},v,t\right)=\varrho_{0}\;\delta \left(v\right).$$

Substitution of Equations (69), (70), and (71) into Equation (67) gives

(72) $$\begin{array}{cc} \langle \mu_{\Gamma },\varphi \rangle =\int_{1}^{\infty }[ & \frac{\varrho_{0}}{\sqrt{2}{\left(1+t\right)}^{3/2}}\;\varphi \;\left(t,s\left(t\right),\frac{\sqrt{2}}{\sqrt{1+t}}\right)\\ \; & +\frac{\varrho_{0}}{\sqrt{2(1+t)}}\;\varphi \left(t,s(t),0\right)]dt. \end{array}$$

Equivalently,

(73) $$\mu_{\Gamma }=\mu_{\Gamma ,L}+\mu_{\Gamma ,R},$$

with

(74) $$\mu_{\Gamma ,L}\left(t,x,v\right)=\frac{\varrho_{0}}{\sqrt{2}{\left(1+t\right)}^{3/2}}\delta \left(x-s\left(t\right)\right)\delta \;\left(v-\frac{\sqrt{2}}{\sqrt{1+t}}\right),$$

and

(75) $$\mu_{\Gamma ,R}\left(t,x,v\right)=\frac{\varrho_{0}}{\sqrt{2(1+t)}}\delta \left(x-s\left(t\right)\right)\delta \left(v\right),\;t\geq 1.$$

**Figure 4:**
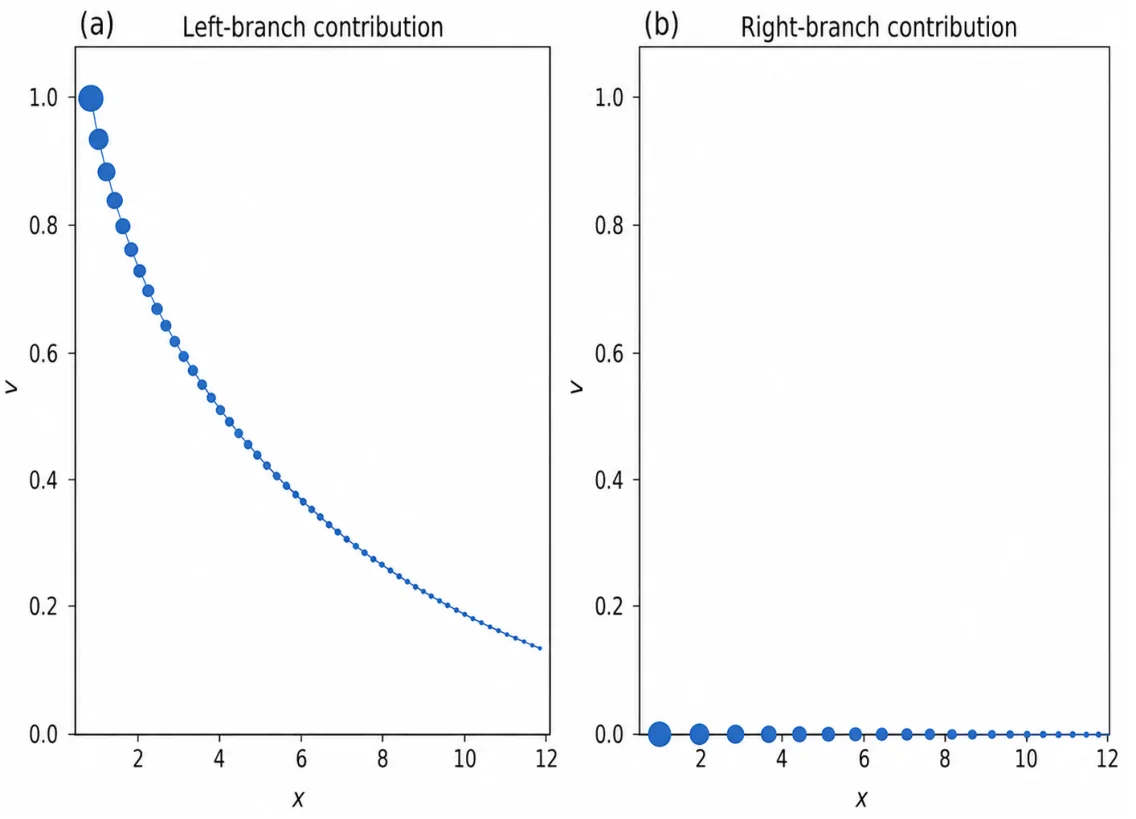
Support and relative weight of the two branch contributions to the shock-supported defect $\mu_{\Gamma }$ for the triangular Burgers benchmark in the (x,v) plane. Panel (**a**) shows the incoming left-branch contribution and panel (**b**) the stationary right-branch contribution. In both panels, marker area schematically indicates relative weight. The full defect is the sum of the two contributions.

The left contribution lies on x=s(t) with $v=u_{L}\left(t\right)$, so in the (x,v) plane its support traces v=2/(x+1). The right contribution lies on the same shock curve in *x* but remains at v=0. Their relative weights decay as ${\left(1+t\right)}^{-3/2}$ and ${\left(1+t\right)}^{-1/2}$, respectively. Figure 4 shows these two support components.

The triangular benchmark therefore supplies a closed-form, time-dependent exact example of the branchwise interface mechanism. In 3D the defect is supported on a moving surface and weighted by the normal relative transport flux; here it is supported on the shock curve and weighted by the branch velocity relative to the shock.

## 5. Discussion

The present construction separates two levels of the KvN/Weyl/Wigner/Moyal description. On an individual smooth Euler branch, the Wigner object evolves under the Moyal equation generated by the branch symbol $H=v\cdot u+h-{\left|u\right|}^{2}/2$. For a general nonlinear branch, higher Moyal terms remain. The Poisson component nevertheless has a direct transport interpretation, and it is this component that produces the explicit interface contribution when two smooth branches are assembled across a moving shock surface.

The interface defect does not represent a new physical source of mass or momentum. Rather, it records the interface-supported distribution required to join the two one-sided smooth phase-space transports. Its coefficient is the normal branch flux relative to the shock speed. Therefore, the same kinematic quantity from the Rankine–Hugoniot balance controls the phase-space defect. While fluid jump and entropy conditions still select the admissible one-sided states and shock motion, the KvN/Weyl/Wigner/Moyal construction records these data in a branchwise phase-space balance. It does not replace the classical admissibility theory.

The exact planar constant-state example makes this interpretation especially transparent. The common shock-frame mass flux transfers between two distinct monokinetic velocity supports. Because no net mass is created at the interface, its zeroth velocity moment vanishes. However, its first moment records the pressure jump already imposed by the Euler momentum condition. The defect can thus be nonzero as a distribution in phase space even when its lowest moment vanishes. This distinction between conservation in physical space and redistribution between velocity supports highlights a key advantage of the phase-space representation.

The Burgers benchmark exhibits a different moment structure because ϱ is an auxiliary transported density rather than the conserved Euler mass density. The left and stationary right traces are both incoming relative to the moving shock, producing support at $v=u_{L}\left(t\right)$ and v=0, respectively. Consequently, the zeroth velocity moment of $\mu_{\Gamma }$ is nonzero and records the accumulation of auxiliary branch weight at the shock. This observation does not introduce a new physical mass or momentum law for Burgers flow; such an interpretation would require an explicitly coupled density–momentum system and a shock-supported density measure.

The planar and Burgers examples are complementary. The planar example features a genuine 3D Euler shock with constant branches, giving an exact branchwise realization of the velocity-support transfer shown in Figure 1. Conversely, the triangular Burgers example provides a one-dimensional scalar benchmark with an auxiliary transported density. It supplies a time-dependent, closed-form case where the shock trajectory, the two branch weights, the associated branchwise Wigner transforms, and both components of the interface defect can all be followed analytically. Together, these examples demonstrate that the localization mechanism is neither an artifact of a stationary planar construction nor a feature confined to scalar Burgers flow. They also distinguish the general result, which retains smooth-branch higher-order Moyal derivative contributions, from the exact quadratic-symbol cases where the Moyal equation reduces to Poisson transport.

The branchwise construction does not identify the assembled object with the exact global Wigner transform of a discontinuous wavefunction formed by joining the two one-sided branches across the interface. A global Wigner transform is nonlocal and can contain cross-interface interference terms whose form depends on how the one-sided wavefunctions extend away from the shock. The present formulation instead isolates the one-sided Wigner traces and the local interface defect generated by their piecewise assembly. This restricted viewpoint suffices for the weak transport identity derived here, but a fully global theory would have to incorporate those nonlocal terms together with any additional singular distributions generated by higher Moyal orders.

The present construction is limited to a formal branchwise weak identity for regular moving shocks with specified one-sided traces. It is neither a general weak-solution theorem nor a closure or shock-selection principle. Within these limits, the formulation integrates smooth phase-space evolution and singular interface transport into a common KvN/Weyl/Wigner/Moyal framework.

A practical numerical realization need not resolve $\mu_{\Sigma }^{P}$ on a dense six-dimensional Cartesian mesh. Instead, the Euler shock may be obtained by shock-capturing or shock-fitting methods, with $\Sigma_{t}$ represented by a level set, tracked front, or fitted interface. One-sided traces can then be reconstructed and the defect evaluated via quadrature on the moving physical-space surface. For monokinetic traces, the velocity delta supports can be retained analytically or represented by particles; more general traces could employ sparse or adaptive velocity discretizations. Such an implementation would use the phase-space defect as a diagnostic tool for the resolved shock geometry and one-sided states. Developing and testing this strategy lies beyond the scope of the present analytical study.

## 6. Conclusions

We have developed a branchwise KvN/Weyl/Wigner/Moyal formulation for shock-containing, irrotational, barotropic Euler flow. General smooth branches obey the Moyal equation associated with the Euler amplitude–phase symbol. When the one-sided solutions are assembled across a moving shock, the Poisson component of the branchwise balance contains an additional defect term supported on the shock interface.

The exact planar constant-state Euler example demonstrates how the conserved mass flux is redistributed between distinct velocity-space supports. The affine triangular Burgers example provides a closed-form, time-dependent benchmark with two defect components supported at the incoming branch velocity and at zero velocity. These examples complement the general 3D weak identity and clarify the circumstances under which the branchwise Moyal evolution reduces exactly to Poisson transport.

The construction does not replace the Rankine–Hugoniot or entropy conditions, nor does it provide an exact global Wigner transform across a discontinuity. Instead, its value lies in organizing the smooth branch dynamics and the singular interface contribution within a single phase-space formulation, thereby providing a precise diagnostic language for a shock-supported structure in Euler flow.

## Data Availability

No new data were created or analyzed in this sutdy. Data sharing is not applicable to this article.

## Appendix A. Technical Clarification and Exact Branch Wigner Transforms

### Appendix A.1. Relation to the Earlier Physical-Space Koopman–Wigner Realization

Ref. [31] used the physical-space advection symbol

(A1) $$H_{\mathrm{adv}}\left(r,v,t\right)=v\cdot u\left(r,t\right).$$

This symbol and its Weyl-ordered operator correctly describe passive transport of a square-root density by a prescribed velocity field. If ψ=Aexp(iϕ/α) evolves under the operator associated with Equation (A1), its amplitude and phase satisfy

(A2) $$\partial_{t}A+u\cdot \nabla_{r}A+\frac{1}{2}\left(\nabla_{r}\cdot u\right)A=0,$$

and

(A3) $$\partial_{t}\phi +u\cdot \nabla_{r}\phi =0.$$

Equation (A2) is the continuity equation for the transported density $A^{2}$. However, imposing ∇ϕ=u in Equation (A3) gives $\partial_{t}\phi +{\left|u\right|}^{2}=0$ rather than the Euler velocity-potential Equation (4). The physical-space amplitude–phase realization therefore requires the scalar contribution $h\left(\rho \right)-{\left|u\right|}^{2}/2$, giving the Weyl symbol used in Equation (9). For the Burgers reduction this becomes $H_{B}=vu-u^{2}/2$.

A second correction concerns the Moyal reduction. Affinity of $H_{B}$ in v alone does not eliminate mixed higher derivatives when the spatial coefficients are nonlinear. Exact Moyal-to-Poisson reduction for arbitrary Wigner symbols requires the Weyl symbol to be at most quadratic in the canonical variables. The constant-state planar branches and the affine Burgers branches used in this paper satisfy that condition.

### Appendix A.2. Planar Constant-State Euler Branches

For a constant branch, choose the global plane-wave extension

(A4) $$\psi_{\pm }\left(r,t\right)=\sqrt{\rho_{\pm }}\exp\;\left[\frac{i}{\alpha }\left(u_{\pm }\cdot r-\omega_{\pm }\left(t\right)\right)\right],$$

where the spatially uniform phase $\omega_{\pm }\left(t\right)$ does not affect the Wigner transform. Substitution into Equation (10) gives

(A5) $$\begin{array}{cc} W_{\pm }\left(r,v,t\right) & =\rho_{\pm }\int \frac{d^{3}y}{{\left(2\pi \alpha \right)}^{3}}\exp\;\left[-\frac{i}{\alpha }\left(v-u_{\pm }\right)\cdot y\right]\\ \; & =\rho_{\pm }\delta^{\left(3\right)}\left(v-u_{\pm }\right), \end{array}$$

which is Equation (37). This is an exact Wigner identity for the specified global plane-wave extension, not for a spatially truncated wavefunction.

### Appendix A.3. Global Affine Extension of the Incoming Burgers Branch

For the convenience of the reader, recall that on the global affine extension of the incoming branch,

(A6) $$u_{L}^{\mathrm{ext}}\left(x,t\right)=\frac{1+x}{1+t},\;\varrho_{L}^{\mathrm{ext}}\left(x,t\right)=\frac{\varrho_{0}}{1+t}$$

(Equation (63)). A potential satisfying both $\partial_{x}\phi_{L}=u_{L}^{\mathrm{ext}}$ and $\partial_{t}\phi_{L}+{\left(u_{L}^{\mathrm{ext}}\right)}^{2}/2=0$ is

(A7) $$\phi_{L}\left(x,t\right)=\frac{x+x^{2}/2+1/2}{1+t}+C_{0},$$

where $C_{0}$ is constant. The corresponding globally extended affine-branch wavefunction is

(A8) $$\psi_{L}^{\mathrm{ext}}\left(x,t\right)=\sqrt{\frac{\varrho_{0}}{1+t}}\exp\;\left[\frac{i}{\alpha }\frac{x+x^{2}/2+1/2}{1+t}\right].$$

Its Wigner transform is

(A9) $$W_{\psi ,L}\left(x,v,t\right)=\int \frac{dy}{2\pi \alpha }\exp\;\left(-\frac{i}{\alpha }vy\right)\psi_{L}^{\mathrm{ext}*}\;\left(x-\frac{y}{2},t\right)\psi_{L}^{\mathrm{ext}}\;\left(x+\frac{y}{2},t\right).$$

The phase difference is exactly

(A10) $$\phi_{L}\;\left(x+\frac{y}{2},t\right)-\phi_{L}\;\left(x-\frac{y}{2},t\right)=\frac{1+x}{1+t}y.$$

Therefore,

(A11) $$\begin{array}{cc} W_{\psi ,L}\left(x,v,t\right)\; & =\frac{\varrho_{0}}{1+t}\int \frac{dy}{2\pi \alpha }\exp\;\left[-\frac{i}{\alpha }y\left(v-\frac{1+x}{1+t}\right)\right]\\ \; & =\frac{\varrho_{0}}{1+t}\delta \;\left(v-\frac{1+x}{1+t}\right), \end{array}$$

which is Equation (68). Because $H_{B}=vu-u^{2}/2$ and *u* is affine, this Wigner function also satisfies Equation (60) exactly. The result refers to the global affine extension used to define the branchwise trace; multiplying the wavefunction by a spatial cutoff before taking the Wigner transform would produce additional nonlocal terms.

For the stationary right extension, $\psi_{R}\left(x,t\right)=\sqrt{\varrho_{0}}$, and the same Wigner convention gives

(A12) $$W_{\psi ,R}\left(x,v,t\right)=\varrho_{0}\;\delta \left(v\right),$$

in agreement with Equation (64).
